# A genetic linkage map for a Full sib population of *Eucalyptus grandis* using SSR, DArT, CG-SSR and EST-SSR markers

**DOI:** 10.1186/1753-6561-5-S7-P26

**Published:** 2011-09-13

**Authors:** Martín García, Pamela Villalba, Cintia Acuña, Javier Oberschelp, Leonel Harrand, Mauro Surenciski, María Martínez, César Petroli, Carolina Sansaloni, Danielle Faria, Dario Grattapaglia, Susana Marcucci Poltri

**Affiliations:** 1 Instituto Nacional de Tecnología Agropecuaria. Instituto de Biotecnología. De Los Reseros y Dr. Nicolás Repetto s/n°, CP 1686, Hurlingham, Buenos Aires, Argentina; 2 EEA INTA Concordia. Grupo de Mejoramiento Genético Forestal. Estación Yuquerí s/n, CP 3200, Concordia, Entre Ríos, Argentina; 3 Embrapa Recursos Genéticos e Biotecnologia - Parque Estação Biológica - PqEB - Av. W5 Norte (final). Caixa Postal 02372, 70770-970 - Brasília, DF – Brazil

## Background

*Eucalypts* are the most widely planted hardwood trees in the world, occupying globally more than 18 million hectares, as an important source of carbon neutral renewable energy and raw material for pulp, paper and solid wood. Intensive planting programs of *Eucalyptus grandis* have been carried out in the Argentinian Mesopotamia.

Linkage maps are useful tools for quantitative trait loci (QTL) analyses and detection. Several maps for QTL analyses of growth and wood quality have been developed in this genus [1,2] and most of the *E. grandis* maps have been carried out in interspecific crosses.Improved marker density in genetic maps, high-throughput techniques and transferability across species are key aspects to increase resolution and speed for a variety of genomic applications in *Eucalyptus*.In this context, an important issue in association studies is the selection of appropriate mapped candidate genes that co-localize with QTL of interest.

As part of the Biotech MERCOSUR project (Marcucci et al., this journal), we here report the construction of a genetic linkage map for *E. grandis* in the context of a QTL study of this specie in an effort to understand the molecular basis for quantitative trait variation in wood quality*.* This map includes Diversity Arrays Technology (DArT) [3], microsatellite (SSR) markers [4], Candidate Genes-SSR (CG-SSR) for wood quality traits and stress responses functions and Expressed Sequence Tag-SSR (EST-SSR) for putative function related to stress responses and other functions (Acuña et al., this journal). These CG-SSR and EST-SSR were not mapped in *Eucalyptus* previously.

## Material and methods

### Plant material

*E. grandis* x *E. grandis* (EG-INTA-161 x EG-INTA-152) F1 population of 130 full-sib progeny cloned (3 ramets) and planted in 2007 in Entre Ríos, Argentina, was analyzed.

### Genotyping

The parents of the mapping cross were initially screened with: 55 SSR, 12 CG-SSR and 37 EST-SSR markers; these last two classes of markers derive from a broad study (for details see Acuña et. al., this journal). Capillary electrophoresis and fluorescent detection were carried out on an ABI 3130xlGenetic Analyzer. A DArT Microarray of 7,860 clones was screened for useful polymorphic markers.

### Linkage and bioinformatic analysis

All loci were tested for goodness of fit to expected Mendelian segregation ratios using Chi-square goodness of fit tests. The assignment of DArT sequence function was performed using the Blast2GO software [http://www.blast2go.org/]. A consensus genetic linkage map was constructed with JoinMap v3.0 [5]. Linkage parameters were set as 10 minimum LOD and 0.4 maximum recombination fractions.

## Results and discussion

In this intraspecific *E. grandis* population, 78% of the SSR markers tested could be mapped. Most mapped SSR loci were fully informative, segregating in approximate ratios of 1:1:1:1 (either heterozygous in both parents four alleles in total). Seven SSR loci that followed approximate segregation ratios of 1:1 (heterozygous in only one parent) were EMBRA 47,51,101,179,676,1244,2010.

From the DArT Microarray of 7,860 clones, 31% of the marker were selected because of their high call rate (>0.80) and polymorphism between parents.

A large proportion (1,503/2,381=63%) of the DArT markers displayed a Mendelian behavior indicating that they sample single copy regions and provide markers that can be used for genetic analyses (65% segregating in 1:1 ratio and 35% in 3:1 ratio).

The map was assembled with 1032 markers, including 976 DArT, 43 SSR loci (2-6 per linkage group), seven EST-SSR and six CG-SSR. The resulting integrated map featured the expected 11 major linkage groups, yielding a genome coverage of 1358.4 cM, and an average consecutive intermarker distance of 1.3 cM in accordance to other reports [1,4]. Linkage groups were numbered following the standardized nomenclature for Eucalyptus proposed by Brondani et al [4].

The six Candidate Genes and seven ESTs include enzymes involved in lignin and cell-wall polysaccharide biosynthesis and stress responses genes, while 267 DArT (29.7%) were assigned to a gene ontology (GO) categories and 296 loci (32.9%) had significant matches with the nonredundant protein database using BLASTX. Thus, 25 enzymes in 56 metabolic pathways were represented by at least one sequence with its corresponding EC number.

The inclusion of common previously mapped SSR markers in several different eucalypt species within the subgenus *Symphyomyrtus* (*E. globulus*, *E. camaldulensis*, *E. dunnii*, *E. tereticornis*, and predominantly *E. grandis* and *E. urophylla*) allowed comparison of linkage groups among this *E. grandis* population and other species of the genus. Linkage orders previously reported in *E. globulus*, *E. grandis* and *E. urophylla* were also observed in this intraspecific *E. grandis* population, supporting the developed map.

## Conclusions

In this work, 13 new functional SSR and 976 DArT (296 with assigned functions) markers are mapped in an intraspecific *E. grandis* F1 population. This map will be used to locate QTL for wood quality and growth traits in the specie*.* Also, this map can help to identify candidate genes and regions in the *Eucalyptus* genome useful for fine scale analysis with association studies that are being developed by our group (see Cappa et al., this volume).The putative functional approach combined with the genetic linkage mapping provides an advantage tool for future analysis in locating genes of interest in *Eucalyptus*.
